# Using Grey Relational Analysis to Evaluate Energy Consumption, CO_2_ Emissions and Growth Patterns in China’s Provincial Transportation Sectors

**DOI:** 10.3390/ijerph14121536

**Published:** 2017-12-08

**Authors:** Changwei Yuan, Dayong Wu, Hongchao Liu

**Affiliations:** 1 School of Economics and Management, Chang’an University, Xi’an 710064, China; changwei@chd.edu.cn; 2 Department of Civil, Environmental and Construction Engineering, Texas Tech University, Lubbock 79409, TX 79409, USA; Hongchao.Liu@ttu.edu

**Keywords:** Grey Relational Analysis, energy consumption, CO_2_ emissions, Chinese transport sector, province-level, sustainable policy

## Abstract

The transportation sector is a complex system. Collecting transportation activity and the associated emissions data is extremely expensive and time-consuming. Grey Relational Analysis provides a viable alternative to overcome data insufficiency and gives insights for decision makers into such a complex system. In this paper, we achieved three major goals: (i) we explored the inter-relationships among transportation development, energy consumption and CO_2_ emissions for 30 provincial units in China; (ii) we identified the transportation development mode for each individual province; and (iii) we revealed policy implications regarding the sustainable transportation development at the provincial level. We can classify the 30 provinces into eight development modes according to the calculated Grey Relational Grades. Results also indicated that energy consumption has the largest influence on CO_2_ emission changes. Lastly, sustainable transportation policies were discussed at the province level according to the level of economy, urbanization and transportation energy structure.

## 1. Introduction

China faces the dynamics of rapid economic development that are driving ever increasing energy use and CO_2_ emissions [1]. According to the International Energy Agency (IEA), China’s energy-related CO_2_ emissions grew by 50% between 1990 and 2000, and doubled from 2000 to 2010, reaching 7 billion tons of CO_2_ in 2010 [2]. Since 2008, China has become the world’s largest CO_2_ emitter, accounting for about one fourth of global greenhouse gas (GHG) emissions. To tackle the severe environmental stress and curb global warming, China made a pledge to reduce its CO_2_ emissions per unit of GDP (known as carbon intensity) by 40% below 2005 levels by 2020 and increase the share of non-fossil fuels in primary energy consumption to around 15% by 2020.

According to the IEA, the transportation sector consumes nearly half of global oil and contributes one quarter of total fossil fuel combustion-related CO_2_ emissions that primarily involve fuel being burned for road, rail, air and marine transportation [3]. In this context, reducing CO_2_ emissions from the transportation sector is gaining in more and more importance, especially as China moves to bring industrial emissions under control. In addition to climate benefits, urban transportation emissions mitigation also provides co-benefits of urban air quality on human health and tropospheric ozone production. Therefore, China’s any attempt to address CO_2_ emission-related environmental issues should pay attention to its transportation sector.

**Figure 1:**
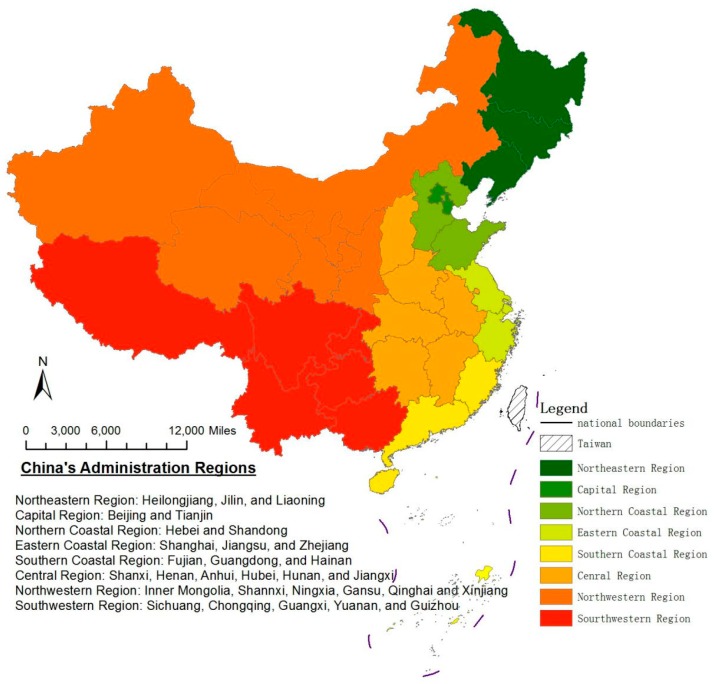
China’s Eight Administration Regions (Northeastern, Capital, Northern Coastal, Eastern Coastal, Southern Coastal, Central, Northwestern, and Southwestern).

However, China differs greatly in geography across the country. Most of China’s coastlines, with a total length of 32,000 km are concentrated in the most eastern ten provincial units, while, with the exception of Guangxi, all of the other western provinces and six central provinces are practically land-locked, as shown in Figure 1. Besides considerable distance from the coast, the combination of high elevation and extremely mountainous topography of central and western provinces puts them in an even worse situation for the construction of transportation infrastructure. Due to such a geographic feature diversity, the early China’s Open-Door policy started from the two southeast coastal provinces in 1978, Guangdong and Fujian, since they are well positioned to offer their port facilities for sea-based transportation routes to foreign investors that seek to import and export their products and manufactured goods overseas. Throughout the 1980s and the early 1990s, the entire country gradually opened to foreign investments and trade, expanding from these two provinces to other coastal areas and spreading further to some central and even western provinces.

**Figure 2:**
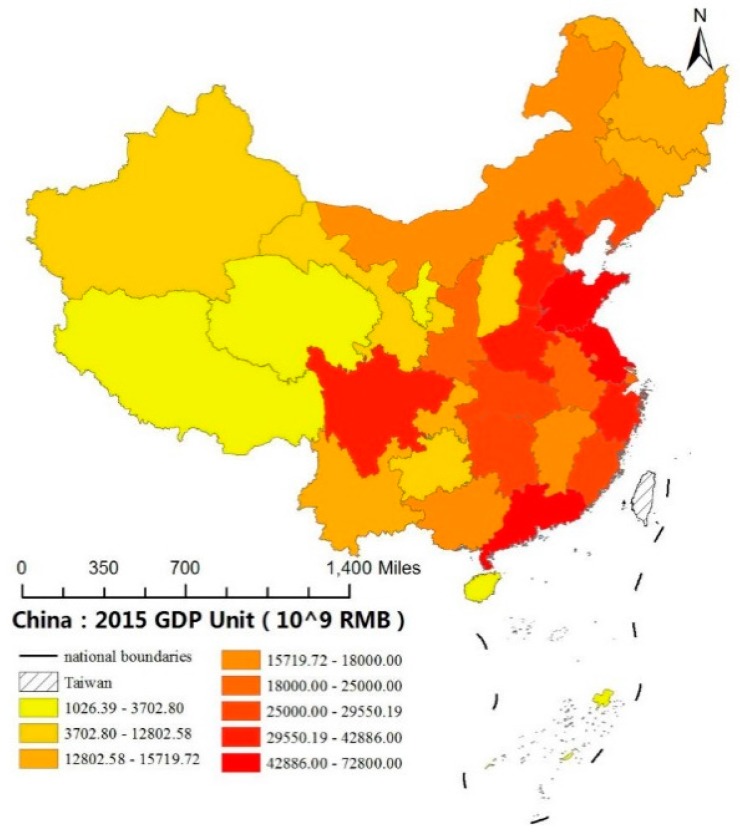
China’s Provincial GDP in 2015; (Note: Ren Ming Bi (RMB), is the Mandarin ping yin (spelling) for China Yuan (CNY). This study includes total 30 Provinces and Municipalities of China, excluding Hong Kong, Macau, Taiwan, and Tibet).

The progression of the launch of the Open-Door policy has created policy advantages for the coastal provinces. The study shows that in the next two decades after the Open-Door policy, the national GDP grew rapidly at an average annual rate of 9.6%, with the eastern, central and western regions growing at 12.8%, 9.3% and 8.7%, respectively [4]. As such, on average, the eastern region was growing at a rate of 4.1% faster than the western region, thereby the gap between the east and the west has widened greatly (for another look at China’s provincial GDPs, see Figure 2). After having been neglected in development and investment for many years, since 2000 the central government’s focus has shifted to the western provinces through China’s Great Western Development Strategy to attract foreign investment to the west and stimulate the economy in that region. As a result the western region has reported an annual average economic growth rate of 10.6% for six years in a row after the implementation of the “go-west” policies [5]. In the 12th Five-Year Plan (2011–2015), the central government continued to prioritize the development of the west and intensify support for Tibet, Xinjiang and other ethnic minority regions. This marked a new round of China's efforts to narrow the development gap between the west and the east.

In the context of these geography and economic variations, the study on China’s CO_2_ emissions should take care of the concerns raised from the diversity of economy and geography at the provincial level. In this regard, although existing studies have made great contributions to CO_2_ emissions in China ([6,7,8]), several important questions have not been answered yet:

- What are the relationships between energy consumption and CO_2_ emissions in China’s transport sector at the regional and provincial levels?
- What are the growth patterns of economy, energy and environment (3E) systems for the Chinese provincial transport sectors?
- What are the impacts of interprovincial inequality in economic development and construction of transportation infrastructure on the 3E’s growth pattern over time?
- How the growth disparities of CO_2_ emissions among eastern, central and western provincial units could be contributed to geography, economy production, energy consumption and transportation activities, respectively?
- Considering the wide variations in the 3E system growth patterns across provincial regions, what clean energy policies should be adopted to mitigate CO_2_ emissions on the province-by-province basis?

This paper makes an effort to address these questions by utilizing Grey Relational Analysis (GRA) to: (i) explore the inter-relationships among transportation development, energy consumption and CO_2_ emissions of 30 provincial units in China; (ii) to further identify the provinces’ development modes in transportation, and (iii) to reveal policy implications regarding sustainable transportation development at the provincial level.

The rest of the paper organizes as follows: Section 2 has three parts: the first part reviews Grey System Theory (GST) and the related grey relational studies; the second part focuses on the methodology of grey relational analysis (GRA) and the concepts of grey relational grade (GRG); and the third part collects and consolidates the China’s 3E systems data at the provincial level from 1995 to 2012. Section 3 presents the GRG results from the GRA and also discusses how to understand them. Section 4 is divided into two sub-sections. The former discusses the GRA results comprehensively, and further classifies the 30 provinces and municipalities into eight development modes based on the GRGs. The latter reveals the policy implications underneath these grey relationships at the provincial level for China’s transportation sector. Finally, we conclude the paper in Section 5.

## 2. Materials and Methods

Grey system theory (GST) was first proposed by Deng [9]. It has been applied widely in the past four decades. In GST, a system with absolutely explicit information is defined as a “white” system. In the contrary, a system with totally unknown information is defined as a “black” system. Most of systems in the real world are thus defined as grey systems that are somewhere between the white and the black, which means that the information and messages of these systems is partially clear. One of the major advantages of GST is that it can generate satisfactory outcomes using a relatively small amount of data or with great variability in the factors since it can increase the data regularity with proper data treatment [10]. Usually, under such a condition (or for a grey system with insufficient information), the results generated by conventional statistical techniques may not be acceptable without sufficient data to achieve desired confidence levels.

Along this line, based on the combined effects of system theory, space theory, and control theory, Deng further proposed the concept of grey relational space to capture the correlations between the reference factor and other factors of a system [11]. The degree of influence of a compared factor on the reference factor, grey relational grade (GRG), can be represented by the relative distance between them in an imaging grey space without making prior assumption about the distribution type. The smaller the distance, the larger the influence. One of the major advantages of grey relational analysis (GRA) is that both qualitative and quantitative relationships can be identified among complex factors with insufficient information of a system.

Over the past three decades, many scholars have discussed the relationship between economic growth, energy consumption and CO_2_ emissions using GRA. For example, Tzeng and Hu employed GRA to calculate the grey relational grades of initial performance indicators for the operation of a bus system in Taiwan [12]. Chang and Chen used GRA to construct evaluation criteria of the grade-separated railroad in the urban area, and analyzed those criteria for transportation, society, economics and technology [13]. Li et al. used GRA to investigate the relationship between the inhibitor structure and inhibitor performance a series of acids causing mild steel corrosion [10]. Chang and Lin identified the key factors affecting CO_2_ emissions from 34 industries in Taiwan by GRA to better understand the inter-relationships regarding industrial environmental, economic and energy performances [14]. Liang used GRA to capture the preliminary schedules for the short-term hydroelectric generation scheduling of a power system and found that hydro schedules generated by the GRA approach were close to the optimal demands by the differential dynamic programming method [15]. Mu et al. applied GRA to explore the relationships among the four major factors that affected the crop residues and firewood consumption in rural household of each province/region in China during 1991–1999 [16]. Lin et al. explored the inter-relationships among economy, energy and CO_2_ emissions of 37 industrial sectors in Taiwan in order to provide insight regarding sustainable development policy making [17]. Several studies have been conducted to forecast how China’s economy will respond to its energy, climate, and environmental policies [18,19,20,21,22,23,24].

Although grey systems theory as a soft science methodology established by Chinese scholars, it has been also widely used by international scholars. For example, Moran et al. presented a new methodology for the evaluation of the environmental and economic feasibility of combustion of different biomass fuels by using GRA [25]. Pai et al. applied grey relation analysis to evaluate the effects of transportation activities on air quality variations in Japan from 1974 to 2004 [26]. In the study of [27], Turkey’s EC and gross domestic product (GDP) relation was analyzed. The relative importance of energy components on GDP for Turkey were found out. Researchers from the UK have proposed a framework for obtaining the optimal electricity generation mix by integrating GRA and multi-objective grey linear programming [28]. The above studies demonstrate that GST and GRA can effectively deal with the system with incomplete and uncertain information such as China’s transport system, using only a few data points.

### 2.1. Introduction of Grey Relational Analysis

GRA is used to explore the qualitative and quantitative relationships among abstract and complex sequences and to capture their dynamic characteristics during the development process. In other words, the calculations of GRA compare the geometric relationships between time series data in the grey relational space (GRS) in terms of the grey relational grade (GRG) ([11,29]). GRG represents the relative variations between one major factor and all other factors in a given system. GRG is large if the relative variations between two factors are basically consistent during their development process, and vice versa.

Let a binary set (X,Γ) denote a GRS, where X is a collection composed of sequences *x_i_* to be compared and reference sequence *x*_0_, Γ is a map set called grey relational map set, *γ*∈Γ is an appointed relational map in GRS. Assume that *γ*(*x*_0_(*k*),*x_i_*(*k*)) is an image at point k from the series to real number with map *γ* and *γ*(*x*_0_,*x_i_*) is an image at all points with *k* = 1, 2, 3, …, n, where: *x*_0_ = (*x*_0_(1),…, *x*_0_(*n*)) and *x_i_* = (*x*_i_(1),…, *x_i_*(*n*)).

Let *γ*(*x*_0_,*x_i_*) satisfy that:

(1) $$\gamma \left(x_{0},x_{i}\right)=\frac{1}{n}\overset{n}{\underset{k=1}{\sum }}\gamma \left(x_{0}\left(k\right),\;x_{i}\left(k\right)\right)$$

Then *γ*(*x*_0_(*k*),*x_i_*(*k*)) is said to be a grey relational coefficient at the point *k* and *γ*(*x*_0_,*x_i_*) to be a grey relational grade, if Γ satisfies the following axioms [11]:

(a) (Norm Interval)

$$\gamma \left(x_{0}\left(k\right),\;x_{i}\left(k\right)\right)\in \left(0,\;1\right],\;\forall k$$

$$\gamma \left(x_{0}\left(k\right),\;x_{i}\left(k\right)\right)=1,\;\mathrm{iff}\;x_{0}\left(k\right)=x_{i}\left(k\right),\;\forall k\;$$

$$\gamma \left(x_{0}\left(k\right),\;x_{i}\left(k\right)\right)=0,\;\mathrm{iff}\;x_{0}\in \varphi ,\;x_{i}\in \varphi$$

where *φ* is an empty set. 

(b) (Duality Symmetric)

$$\gamma \left(x_{0}\left(k\right),\;x_{i}\left(k\right)\right)=\gamma \left(x_{i}\left(k\right),\;x_{0}\left(k\right)\right),\;\mathrm{iff}\;X=\left\{x_{0},x_{i}\right\}$$

(c) (Wholeness)

$$\gamma \left(x_{0}\left(k\right),\;x_{i}\left(k\right)\right)\neq \gamma \left(x_{i}\left(k\right),\;x_{0}\left(k\right)\right)\;\mathrm{almost}\;\mathrm{always},\;\mathrm{iff}\;X=\left\{x_{j}|j=0,1,2,\;\ldots ,n,\;n>2\right\}$$

(d) (Approachability)

$\gamma \left(x_{0}\left(k\right),\;x_{i}\left(k\right)\right)$ decreases along with Δ(k) increasing, where:

$$\Delta \left(k\right)={\left[{\left(x_{0}\left(k\right)-x_{i}\left(k\right)\right)}^{2}\right]}^{\frac{1}{2}}$$

Then, we have a following expression for *γ*(*x*_0_(*k*), *x_i_*(*k*)) which satisfies all the above axioms:

(2) $$\gamma \left(x_{0}\left(k\right),\;x_{i}\left(k\right)\right)=\left(\Delta_{min}+\rho \Delta_{max}\right)/\left(\Delta_{0i}\left(k\right)+\rho \Delta_{max}\right)$$

where: $\Delta_{0i}\left(k\right)=\left|x_{0}\left(k\right)-x_{i}\left(k\right)\right|;\;\Delta_{min}=\underset{i}{\min}\underset{k}{\min}\left|x_{0}\left(k\right)-x_{i}\left(k\right)\right|;\;\Delta_{max}=\underset{i}{\max}\underset{k}{\max}\left|x_{0}\left(k\right)-x_{i}\left(k\right)\right|$.

*ρ*∈(0,1) is a distinguishing coefficient used to adjust the range of the comparison environment, and to control level of differences of the relational coefficients. When *ρ* = 1, the comparison environment is unaltered; when *ρ* = 0, the comparison environment disappears. According to the sensitivity analysis of [14], we adopt *ρ* as 0.5 to perform the GRA because this value offers moderate distinguishing effects and good stability.

At last, we can get the grey relational grade by calculating the average value of all grey relational coefficients *γ*_0i_:

(3) $$\gamma_{0i}=\gamma \left(x_{0},\;x_{i}\right)=\frac{1}{n}\overset{n}{\underset{k=1}{\sum }}\gamma \left(x_{0}\left(k\right),\;x_{i}\left(k\right)\right)$$

The GRG represents the relative variations between two factors indicating magnitude and gradient in a given grey system [13]. If the relative variations of two factors are basically consistent in development trend, then the absolute value of GRG between them is close to 1. However, the specific trend of development among factors cannot be recognized only by the absolute value of GRG. For example, if the GRG between transport turnover and CO_2_ emissions is 0.90, transport turnover may grow faster than CO_2_ emissions from the transport sector, or in a contrary direction. To address this issue, we have adopted the sign conventions of GRGs proposed by Lin et al. [17]. As shown in Equation (4), a positive value of *α* indicates that the growth rate of the compared series is faster than the reference series. For a negative value of *α*, the opposite trend holds. The grey relational grade with direction, *G*_0*i*_ is finally defined as:

(4) $$G_{0i}=\alpha \cdot \gamma_{0i}$$

### 2.2. Grey Relational Analysis in Transportation Sector

By using the GRA and the related GRGs, we can identify the development characteristics of the transport sector. Usually, Carbon Intensity (*CI*) can be defined by Equation (5):

(5) CI=EI×EC

where: *CI* = Carbon Intensity = CO_2_/GDP; *EI* = Energy Intensity = Energy/GDP; *EC* = Emission Coefficient = CO_2_/Energy. *CI* denotes the level of CO_2_ emission per unit of economic output; *EI* denotes the level of energy consumption per unit of economic output; *EC* denotes the level of CO_2_ emission per unit of energy consumption. Since our study is focused on the transportation sector, we need to make an adaption to Equation (5). Let’s substitute GDP with the total transport turnover, then the form of Equation (5) will be changed as:

(6) $$CI_{T}=EI_{T}\times EC$$

where: *CI_T_* = Carbon Intensity in Transport = CO_2_/Total Transport Turnover; *EI_T_* = Energy Intensity = Energy/Total Transport Turnover; *EC* = Emission Coefficient = CO_2_/Energy. *CI_T_* denotes the level of CO_2_ emission per unit of transport output; *EI_T_* denotes the level of energy consumption per unit of transport output; *EC* is the same term as used in Equation (5).

### 2.3. Data Consolidation

In this study, our main objective is to identify the key factors affecting CO_2_ emissions in China’s transport sector at the provincial level and further to differentiate the development mode for each province. Thus, we choose CO_2_ emission data as the reference series, and two transportation-related factors, namely total transport turnover and total energy consumption, as the compared series. Energy consumption data are adopted from energy balance tables for each province in previous China Energy Statistical yearbooks [30]. The fuel types include coal, gasoline, kerosene, diesel, fuel oil, liquefied petroleum gas, natural gas, heat and electricity. Noted that we assume that heat and electricity do not directly generate carbon emissions, which is consistent with most studies [21]. To ensure comparability among the data, energy sources are converted into standard coal equivalent according to standard coal coefficients from Appendix 4 of the 2013 China Energy Statistical Yearbook. Carbon emission coefficients upon the standard coal equivalent are referred to Zhao [31]. Then, following the Intergovernmental Panel on Climate Change (IPCC) Guidelines for National Greenhouse Gas Inventories [32], the CO_2_ emissions are estimated by type of fuel and mode, using the corresponding fuel consumption and associated emission factors.

Transport turnover data are collected from the Yearbooks of China Transportation and Communications [33]. It should be noted that transport turnover data is measured by ton kilometers (ton-km). In this regard, the turnover of passenger-trips that is measured by person-km should be converted to the data in ton-km. The converted turnover of passenger and freight traffic is equal to the turnover of passenger traffic multiplied by a conversion coefficient (0.1), plus the turnover of freight traffic [8]. The conversion coefficient is determined by comparing revenues and expenditures per person-km (or moving one person 1 km) with those of moving one ton of goods 1 km, based on the Transportation Enterprise Cost Management Method issued by the Chinese Ministry of Finance and the Chinese Ministry of Transportation.

## 3. Results 

According to the GRA, the GRGs of two compared factors (total transport turnover and total energy consumption) versus CO_2_ emissions are calculated to explore the inter-relationships among energy consumption, transportation development and CO_2_ emissions for each provincial unit. Let’s denote G_CE_ as the GRG between total energy consumption and CO_2_ emissions, and G_CT_ as the GRG between total transport turnover and CO_2_ emissions. The calculated GRGs are summarized in Table 1. In next section, we will provide thorough discussions on these GRGs.

Next, we will provide some examples about understanding the GRGs in Table 1. Three cases are to be discussed: the case with negative GRGs, the case with positive GRGs, and the case with mixed GRGs.

### 3.1. The Case with Negative GRGs

If we highlight Beijing in Table 1, the values of G_CE_ and G_CT_ for Beijing are −0.9768 and −0.6802, respectively. The negative values indicate that the growth of the two compared factors are slower than that of CO_2_ emissions. According to Equation (6), all of three indicators (CI_T_, EI_T_, and EC) have increased during the study period and two of them (CI_T_ and EI_T_) have presented a significant increase trend (denoted by “↑↑” Table 2). The increase of these indicators show that Beijing’s transport sector is heavily depended on high-energy intensive and CO_2_-intensive transport modes or fuels. In addition, since the absolute value of G_CE_ is higher than that of G_CT_, the growth of energy consumption has more significant influence on CO_2_ emission changes.

### 3.2. The Case with Positive GRGs

In this case, we are taking Guangdong as another example. Both of its GRGs, G_CE_ and G_CT_, are positive (0.9896 and 0.6743). This means that the growth rates of energy consumption and transport turnover are higher than that of CO_2_ emissions. Therefore, all of three indicators (CI_T_, EI_T_ and EC) have decreased during the study period and two of them (CI_T_ and EI_T_) presented a significant decrease trend (denoted by “↓↓” Table 2). In other words, the development of transport in Guangdong is decoupled from CO_2_ emissions by the improved emission coefficients and carbon intensity. If considering as a well-developed coastal province in China, Guangdong has made an effective mitigation of CO_2_ emissions while keeping the high development level of its transport sector in the past 15 years. In this regard, the development mode of Guangdong is superior to that of Beijing.

### 3.3. The Case with Mixed GRGs

For those provinces with the mixed GRGs, the development trends are also mixed. For example, if looking at Hebei in Table 1, the negative value of G_CE_ (−0.9740) and the positive value of G_CT_ (0.7831) exhibit that high carbon energy structure is the main factor to boost up CO_2_ emissions in Hebei’s transport sector, despite that the development of transport turnover is decoupled from CO_2_ emissions. Another example is Jilin in Table 1, where the positive value of G_CE_ (0.9520) and the negative value of G_CT_ (−0.7619) implies that the energy consumption grew faster than CO_2_ emissions, but transport turnover is slower than the increase of CO_2_ emissions. For the two provinces, the GRGs also indicate that energy consumption is the principal factor for the increase of CO_2_ emissions due to its larger absolute value.

## 4. Discussion

### 4.1. Development Modes in China’s Provincial Transportation Sectors

In the previous section, we have explored the correlations of energy consumption, transport development and CO_2_ emissions for the 30 Chinese provincial units. Based on the above analysis, we further identified the development patterns for these 30 provincial transport sectors and then classified them into totally eight development modes, as summarized in Table 2. These development modes are also depicted in − for a better illustration purpose.

**Figure 3:**
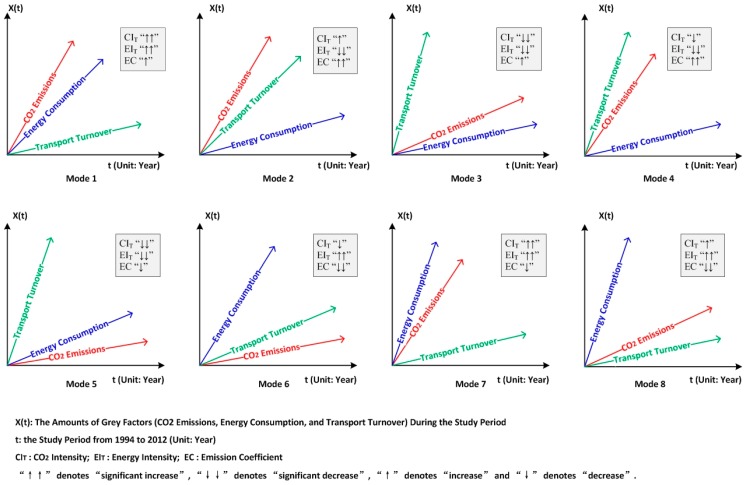
Eight Development Modes of Energy Consumption, CO_2_ Emissions and Transport Turnover in China’s Provincial Transportation Sectors.

Next, we will provide an example to better understand these identified development modes. Firstly, let’s take Mode 4 in Figure 3 as an example. In Mode 4, total transport turnover has the largest growth rate, CO_2_ emissions the second, and total energy consumption the smallest. Such pattern implies that CO_2_ emissions and energy consumption per unit transport turnover have both declined, thus the performance in relation to Carbon Intensity (CI_T_) and Energy Intensity (EI_T_) have been improved. Nevertheless, the fact that the growth rate of CO_2_ emissions exceeded that of energy consumption implies a rise on CO_2_ emission per unit energy consumed, thus the performance regarding CO_2_ emission coefficient has been worsened. The growth patterns of these three factors for other development modes can be explained in a similar way.

After understanding the meaning of each development mode, we can then elaborate the development modes for all 30 provincial units. As observed from Table 2, 11 provincial units (Beijing, Chongqing, Guizhou, Hainan, Heilongjiang, Hubei, Hunan, Qinghai, Shaanxi, Shanxi, and Sichuan) are classified into Mode 1. All of the GRGs are negative values, which implies that the growth of two transportation-related factors are lower than the increase of CO_2_ emissions. This Mode is the worst development scenario since the development of transport sector is coupled with CO_2_ emissions.

In the contrary, Mode 5 presents the best development scenario. The positive GRGs indicate that the growth rates of the two compared factors are higher than that of CO_2_ emissions. This indicates that the development of transport is decoupled from the increase of CO_2_ emission in these provinces. Six provincial units, namely, Guangdong, Henan, Jiangxi, Liaoning, Shanghai, and Tianjin, belong to this group. In Mode 3, the value of G_CT_ is positive and the value of G_CE_ is negative, which implies that the growth of transport turnover is faster than the increase of CO_2_ emission, while the growth of energy consumption is lower than that of CO_2_ emission. Eight provinces, namely, Anhui, Fujian, Gansu, Hebei, Jiangsu, Ningxia, Xinjiang, and Zhejiang, are classified into this group. Similarly, in Mode 7, transport turnover increases slower than CO_2_ emissions (the negative value of GCT), while energy consumption grows faster than CO_2_ emissions (the positive value of GCE). This group has five provinces, namely, Guangxi, Jilin, Inner Mongolia, Shandong, and Yunnan.

**Figure 4:**
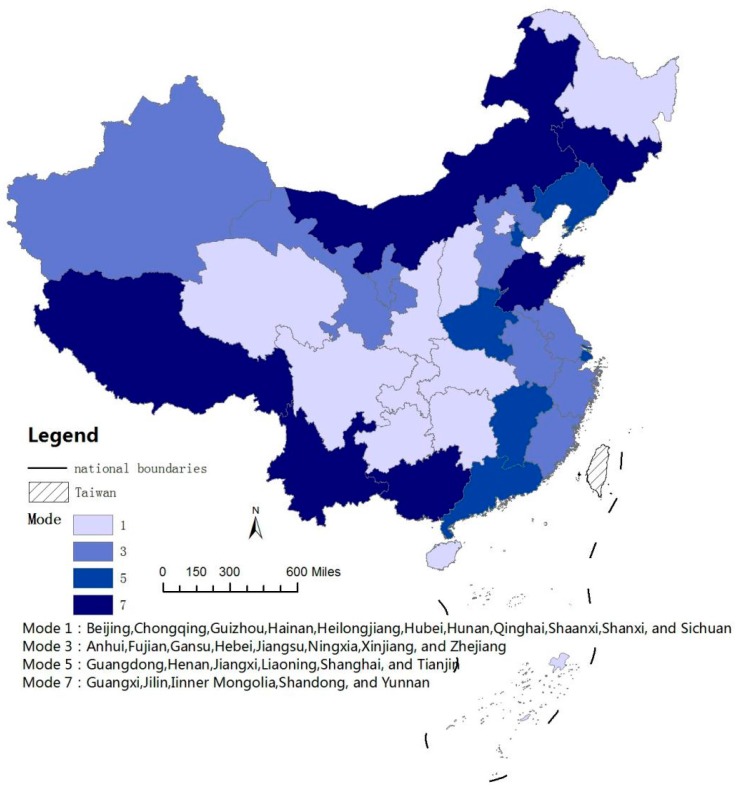
The Transport Development Mode Map for China’s 30 Provinces and Municipalities.

The changing trends of three indicators (CI_T_, EI_T_ and EC) are also presented in Table 2. It should be noted that no provinces have fallen into Modes 2, 4, 6, and 8. Therefore, the development characteristics of these four modes are not elaborated here. Figure 4 shows the transport development mode map for all the studied 30 provinces and municipalities.

### 4.2. Policy Implications

As discussed in the preceding section, transport development modes differ from province to province. For example, Guangdong, Shanghai, Tianjin and other provinces in Mode 5 present decoupling relationships between their transport development and CO_2_ emissions. Energy consumption is also delinked from CO_2_ emissions in their transport sectors. However, the provinces in Mode 1 (e.g., Beijing, Chongqing, etc.) are still under the high pressure of transport-related CO_2_ emissions according to the GRA results. There is a great need for the governments of these provinces to put forth more effective low-carbon transport policies to reverse this development tendency. For other provinces in Mode 3 and Mode 7, mixed development trends appear in their transport sectors. For example, Anhui, Fujian, and other provinces in Mode 3 present the decoupling relationship between their transport development and CO_2_ emissions, while energy consumption is still coupled with CO_2_ emissions in their transport sectors. The provinces in Mode 7 (Shandong, Jilin, etc.) show the contrary trends: transport development is still coupled with CO_2_ emissions, while energy consumption is decoupled from CO_2_ emissions.

In this regard, specific policies need to be provided for different provinces according to their individual development modes. For example, Beijing (the example of the worst development mode) needs to put more emphasis on sustainable policies such as supporting lower emitting vehicles, promoting bio fuels usage, providing and subsidizing public transport, constructing regional carbon trading systems, and promoting the integration of different transport modes (e.g., new bike-sharing systems in Beijing) [34]. Beijing also needs to slow down its rapid growth trend in the private car ownership by implementing policies such as traffic restrictions based on license plate numbers, curbing the purchase of vehicles for private use, increasing fuel tax or parking rates, and even levy carbon tax on transportation if needed.

Another important policy adopted by the Beijing government is promoting electric vehicles (EV) on its road network. The previous study found that Beijing provides the most generous subsides in the purchase of EV [35]. Other than these monetary incentives, Beijing also provides many non-monetary incentives for EV: (1) Buyers of EV can bypass the lottery system for the right to register new vehicles. The odds of winning were only 0.15% in early 2016 for regular vehicle ownerships; (2) EV owners are not subject to a rule banning cars from roads one work-day per week in Beijing.

For Jiangsu Province (where the development mode is Mode 3), transport development is decoupled from CO_2_ emissions in its transport sector. The famous giant West-East Natural Gas Pipeline finished its first phase in 2004, which guaranteed the energy structure adjustment in Jiangsu Province effectively. By decreasing the proportions of coal in the primary energy gradually and increasing the ratio of clean and high-quality energy such as natural gas, solar and electricity, Jiangsu Province realized its energy structure optimization. These changes make it possible for Jiangsu to realize harmony between economic growth and carbon emissions. However, the government of Jiangsu Province still needs to take measures to lower the level of CO2 emissions per unit of energy consumption, since Energy Coefficient still has an increase trend according to the GRA results.

For other central and western provinces (e.g., Shanxi Province in Mode 1), the provincial government should actively seek financial, tax and other preferential policies from the central government to support their low-carbon transport development, such as the incentives to apply new energy technologies in transportation (e.g., natural gas, electricity, and other clean energies) to gradually reduce the proportion of high-polluting energy in total transportation energy consumption, or to improve transport energy efficiency and promote structural optimization of transportation energy, such as a substantial traffic share increase in rail transportation, new energy vehicles and other green modes of transportation, thereby slowing down the increase of transportation emissions. Also, Shanxi Province is the main producer of coal in China. Due to the increasingly intensive freight transportation activity and the associated emissions, the Shanxi Province government should optimize its freight transport planning and organization at provincial or regional levels to reduce the empty-load rate. By increasing the proportion of heavy-lift vehicle, the energy efficiency level of freight transportation can be improved, and hence freight-related emissions should be reduced as well.

For other policies at the national level, it is time for the Chinese government to consider introducing a stiff carbon tax on transportation, and other more robust energy-saving measures and advanced technology incentives. Chinese central and local governments at all levels should improve the allocation of existing transportation special funds, such as increasing the investment of low-carbon transport and new energy-saving technology, promoting low-carbon transformation of existing transport equipment, upgrading existing road networks in Chinese rural areas, and among others.

## 5. Conclusions

In this study, we explored the inter-relationships among transportation development, energy consumption and CO_2_ emissions for China’s 30 provincial units by using the GRA despite insufficient data. Total eight development modes of economy, energy and environment (3E) systems were identified due to the values and signs of GRGs. Among them, Mode 1 presents the worst development scenario due to all negative GRGs, which implies that transport development is closely coupled with CO_2_ emissions. The results also showed that energy consumption has the closest relationship with CO_2_ emission changes. The changing trends of three emission indicators (CI_T_, EI_T_ and EC) were discussed for each province or municipality in the discussion part.

Finally, we revealed policy implications regarding sustainable transportation development. At the technical side, the government should accelerate the development and application of new energy technologies in transportation. On the policy side, a stiff carbon tax on transportation, and other more robust energy-saving measures and advanced technology incentives should be implemented. At the provincial level, specific policies need to be provided for different provinces, due to their different levels of economic development, urbanization and transport structure.

Since building the green transportation system will depend on whether the concern is greenhouse gases like carbon dioxide or hazardous air pollutants such as PM_2.5_, NO_x_, or SO_x_, or most likely, some combination of both. In this regard, reducing air pollutants from the transportation sector will be also an important research topic. PM_2.5_ and NO_x_ also depend on how the vehicle is driven, which is difficult to estimate, and only limited information can be available. In our future work, we will apply the GRA to analyze the trends and driving factors of air pollutants with limited data.

## Figures and Tables

**Table 1:** GRGs between Two Transport-Related Factors and CO_2_ emissions for 30 Provinces and Municipalities of China (1994 to 2012).

Provincial Unit	G_CE_ (Energy Consumption)	G_CT_ (Transportation Turnover)
Anhui	−0.9755	0.7096
Beijing	−0.9768	−0.6802
Chongqing	−0.9608	−0.7836
Fujian	−0.9922	0.8823
Gansu	−0.9367	0.6259
Guangdong	0.9896	0.6743
Guangxi	0.9900	−0.7372
Guizhou	−0.9949	−0.7947
Hainan	−0.9710	−0.6946
Hebei	−0.9740	0.7831
Heilongjiang	−0.9763	−0.7696
Henan	0.9872	0.7982
Hubei	−0.9586	−0.7244
Hunan	−0.9785	−0.7623
Jilin	0.9520	−0.7619
Jiangsu	−0.9862	0.8387
Jiangxi	0.9913	0.6052
Liaoning	0.9689	0.7202
Inner Mongolia	0.9758	−0.8606
Ningxia	−0.9028	0.6220
Qinghai	−0.9613	−0.8760
Shandong	0.9846	−0.7696
Shanghai	0.9845	0.6690
Shaanxi	−0.9681	−0.8623
Shanxi	−0.9562	−0.7971
Sichuan	−0.9710	−0.7716
Tianjin	0.9927	0.6787
Xinjiang	−0.9326	0.7954
Yunnan	0.9900	−0.6747
Zhejiang	−0.9855	0.7739

**Table 2:** The Development Modes of China’s Provincial Transport Sector from 1994 to 2012.

Development Mode	GRG of Transport Turnover G_CT_	GRG of Energy Consumption G_CE_	GRA Comparison	Trend	Provincial Units
Mode 1	<0	<0	|G_CE_| > |G_CT_|	CI_T_ “↑↑” EI_T_ “↑↑” EC “↑”	Beijing, Chongqing, Guizhou, Hainan, Heilongjiang, Hubei, Hunan, Qinghai, Shaanxi, Shanxi, Sichuan,
Mode 2	<0	<0	|G_CE_| < |G_CT_|	CI_T_ “↑” EI_T_ “↓↓” EC “↑↑”	N/A
Mode 3	>0	<0	|G_CE_| > |G_CT_|	CI_T_ “↓↓” EI_T_ “↓↓” EC “↑”	Anhui, Fujian, Gansu, Hebei, Jiangsu, Ningxia, Xinjiang, Zhejiang
Mode 4	>0	<0	|G_CE_| < |G_CT_|	CI_T_ “↓” EI_T_ “↓↓” EC “↑↑”	N/A
Mode 5	>0	>0	|G_CE_| > |G_CT_|	CI_T_ “↓↓” EI_T_ “↓↓” EC “↓”	Guangdong, Henan, Jiangxi, Liaoning, Shanghai, Tianjin
Mode 6	>0	>0	|G_CE_| < |G_CT_|	CI_T_ “↓” EI_T_ “↑↑” EC “↓↓”	N/A
Mode 7	<0	>0	|G_CE_| > |G_CT_|	CI_T_ “↑↑” EI_T_ “↑↑” EC “↓”	Guangxi, Jilin, Inner Mongolia, Shandong, Yunnan
Mode 8	<0	>0	|G_CE_| < |G_CT_|	CI_T_ “↑” EI_T_ “↑↑” EC “↓↓”	N/A

Note: G_CT_: GRG between transport turnover and CO_2_ emission; G_CE_: GRG between energy consumption and CO_2_ emission; CI_T_: CO_2_ intensity; EI_T_: energy intensity; EC: emission coefficient; “↑↑”: significant increase; “↓↓”: significant decrease; “↑”: increase; “↓”: decrease.
